# Epigenetic genome control by heterochromatin and RNAi machinery

**DOI:** 10.1186/1756-8935-6-S1-O32

**Published:** 2013-03-18

**Authors:** Shiv Grewal

**Affiliations:** 1 Laboratory of Biochemistry and Molecular Biology, Center for Cancer Research, National Cancer Institute, National Institutes of Health, Bethesda, Maryland, USA

## 

RNAi is a conserved mechanism in which small interfering RNAs (siRNAs) guide the degradation of cognate RNAs, but also promote heterochromatin assembly at repetitive DNA elements such as centromeric repeats. However, the full extent of RNAi functions and its endogenous targets have not been explored. We show that in the fission yeast *Schizosaccharomyces pombe*, RNAi and heterochromatin factors cooperate to silence diverse loci, including sexual differentiation genes, genes encoding transmembrane proteins, and retrotransposons that are also targeted by the exosome RNA degradation machinery. In the absence of the exosome, transcripts are processed preferentially by the RNAi, revealing siRNA clusters and corresponding increase in heterochromatin modifications across large domains containing genes and retrotransposons. Interestingly, the generation of siRNA clusters and heterochromatin assembly by RNAi is triggered by a mechanism involving the canonical poly(A) polymerase Pla1 and an associated RNA surveillance factor Red1, which also activate the exosome. More importantly, siRNA production and heterochromatin modifications at these target loci are regulated by environmental growth conditions, and by developmental signals that induce gene expression during sexual differentiation. These analyses uncover interplay between RNAi and the exosome that is conserved in higher eukaryotes, and show that differentiation signals modulate RNAi silencing to regulate developmental genes.
